# Pregnancy-Related Hip and Pelvis Musculoskeletal Conditions, Risk Factors, and Prevention

**DOI:** 10.1007/s12178-025-09991-6

**Published:** 2025-08-19

**Authors:** Chloe J. Leung, Frances Tao, Carlin Senter, Faustine D. Ramirez, Stephanie E. Wong

**Affiliations:** 1 Department of Medicine, University of California, Los Angeles, San Luis Obispo, CA USA; 2 Department of Orthopaedic Surgery, University of California, San Francisco, 1500 Owens St, Suite 170, San Francisco, CA 94114 USA

**Keywords:** Pregnancy, Hip, Pelvis, Musculoskeletal conditions

## Abstract

**Purpose of Review:**

This review article examines the etiology, treatments, and outcomes related to common hip and pelvis musculoskeletal conditions associated with pregnancy. We explore the physiological changes that occur during pregnancy and other risk factors to better understand these conditions and their management.

**Recent Findings:**

While some physiological changes are inevitable, other behavioral and psychological factors can be modified in ways that promote healthy pregnancies and better birth and postpartum outcomes.

**Summary:**

We shed light on the potential of pre-habilitation exercises for injury prevention and explore the spectrum of treatment options available to pregnant women including physical rehabilitation, complementary and alternative modalities, and medications.

## Introduction

Musculoskeletal pain is commonly associated with pregnancy and the true prevalence is likely underestimated [1, 2]. This may partially be attributed to the tendency for musculoskeletal pain to be dismissed as typical aches and pains of pregnancy, such that many musculoskeletal conditions remain undiagnosed or misdiagnosed, and thus inadequately managed [2, 3]. One retrospective study estimated that nearly all pregnant women endure some form of musculoskeletal disorder, with nearly 25% becoming temporarily disabled due to symptoms [4].

The musculoskeletal conditions that women experience vary by trimester, with many injuries affecting the hip and pelvis regions. The first trimester is typically characterized by back pain alone, while later trimesters witness the onset of joint effusion, nerve entrapment, as well as worsening back pain, pelvic girdle pain, pubic symphysis pain, and sacroiliac joint pain [5]. Up to 1 in 5 pregnant women experience pelvic girdle pain and over a third complain of hip pain [2, 6]. A retrospective cross-sectional study of nearly 600 women found that greater intensity of pelvic and low back pain during the third trimester were predictors of duration of labor and mode of delivery (unassisted vaginal delivery, assisted vaginal delivery, cesarean section) [7]. Several factors influence the development of these conditions during pregnancy. Important anatomical and physiological changes, in addition to fetal positioning, physical activity during pregnancy, and social determinants of health, put pregnant women at risk for musculoskeletal conditions.

In this review article, we explore the etiology, treatments, and outcomes of pregnancy-related musculoskeletal hip and pelvis conditions with the overarching goal of informing better identification, treatment, and prevention of these conditions, as well as to recommend updating physical activity guidelines for pregnant women.

## Factors Affecting Hip and Pelvic Musculoskeletal Conditions during Pregnancy

### Anatomical and Physiological Changes

Musculoskeletal conditions may develop due to significant changes in hormone levels, weight gain, fluid retention, and nutritional deficiencies that accompany pregnancy. Ligamentous laxity is mediated by altered levels of relaxin and estrogen [8, 9]. These hormonal changes modify the properties of pelvic connective tissue and increase joint movement to prepare the body for the growing fetus and subsequent childbirth [8]. The enlarging uterus weakens abdominal musculature while increasing lumbar strain, thereby shifting the body’s center of gravity, and changing gait and biomechanics in compensation [1, 5]. The resulting adaptations to the structure of the pelvis pose great challenges to the ability to balance, pain levels, and risk for musculoskeletal injury [5]. Weight gain throughout pregnancy increases mechanical stress and load onto joints [5, 8, 10]. Fluid retention, especially during late pregnancy, may result in soft tissue edema and compression of local structures, which predisposes pregnant women to develop tenosynovitis and nerve entrapment [5, 8]. Pregnant women may also experience morning sickness, which has been associated with impaired maternal nutrition [11]. Impaired nutrition could then hinder optimal musculoskeletal function and could delay recovery from injury.

### Position of the Fetus

Malpositioning of the fetus is a potential risk factor for pelvic pain during the late third trimester of pregnancy and delivery [7]. In one retrospective cross-sectional study, 460 women were asked to self-report the position of their fetus before delivery [7]. The researchers categorized occiput anterior as “optimal,” and occiput posterior or occiput transverse as “suboptimal” [7]. Women who had “optimal” positioning of their fetus reported significantly lower levels of pain in the third trimester of pregnancy compared to women who had “suboptimal” positioning [7]. Of note, the fetal positions were not clinically confirmed in this study and thus drawing definitive conclusions remains difficult.

### Social Determinants of Health

A multitude of social determinants of health influence the obstetric care, and plausibly the orthopedic care, that pregnant women receive. Women of low socioeconomic status experience delays in access to reproductive healthcare and are more likely to experience poor family planning outcomes such as unintended pregnancy, unintended births, abortions, and teen pregnancies [12]. Type of employment can also increase a woman’s risk for hip and/or pelvic pain during pregnancy. A systematic review of over 140,000 pregnant women examined the relationship between occupations that require lifting and pregnancy-related musculoskeletal disorders [13]. The researchers discovered that lifting heavy items (i.e., ≥ 22 lbs or ≥ 10 kg) during pregnancy was associated with increased risk of experiencing pelvic girdle pain and requesting early leave from work [13]. A Danish study of 910 pregnant women employees found that poor health status, physical work factors (heavy lifting, shift work), and psychosocial factors (stress, burnout, and depression) were associated with taking more sick leave [14].

### Race and Ethnicity

In the United States, complications of pregnancy affect Black women much more frequently than women of other races. The maternal mortality rate for non-Hispanic Black women was nearly 3 times that of non-Hispanic white and Hispanic women in 2021 [15]. Women of racial minorities may have their pain more frequently dismissed by healthcare providers [16, 17]. A retrospective cohort study of nearly 10,000 women found that postpartum pain management was provided more frequently to non-Hispanic white women during inpatient treatment and upon hospital discharge, despite Hispanic and non-Hispanic Black women reporting greater pain scores [17]. Moreover, historical trauma from being the victims of medical experimentation, discriminatory healthcare practices, and forced sterilization has given rise to mistrust of the medical establishment among racial minorities [18, 19]. All of these factors likely continue to contribute to decreased reporting and identification of painful musculoskeletal conditions among these demographics of pregnant women.

### Physical Activity

Physical activity during pregnancy is associated with lower rates and lower intensities of low back and pelvic pain in pregnant women [20–23]. Physical activity during pregnancy is considered relatively safe, with overall low incidence rates of injury. A systematic review and meta-analysis found that prenatal exercise was not associated with injury or musculoskeletal trauma, and there was no exercise dose-response relationship with labor and delivery outcomes [24]. In a prospective cohort study of over 1,400 pregnant women with varying physical activity levels, 98% reported no physical activity-related injury during pregnancy [25]. The incidence rate of physical activity-related injury was a mere 3.2 per 1,000 h of physical activity, while the injury incidence rate related to exercise was 4.1 per 1,000 exercise hours [25]. Most reported injuries were minor and comprised of bruises, scrapes, strains, or sprains. Most injuries occurred in the ankle (21%), knee (16%), back (14%), abdomen (9%), and tailbone (9%) [25]. The pelvis was affected in 5% of injured participants while the hip was injured in 2% [25]. The majority of injuries (67%) were related to falls while walking through regular activities of daily living that were not associated with exercise [25]. Interestingly, 85% of participants reported engaging in moderate intensity physical activity (3–6 METS) at the time of injury [25]. Light physical activity, including walking, appears to be safe and was not associated with musculoskeletal injury.

Not only is the incidence of pregnancy-related musculoskeletal pain lowered with exercise, but the severity of pain can be improved as well. Select types of exercises and their associated musculoskeletal injuries are highlighted below.

### Running

Running is a common form of aerobic exercise that many women choose to engage in during pregnancy. However, it is not without risk of musculoskeletal pain. A retrospective cross-sectional study of over 3,100 women who ran prior to and during pregnancy revealed that the majority (86%) experienced some form of pain while running [26]. Participants experienced pain in the pelvis/sacroiliac joint (59%), lower back (52%), abdomen (51%), breast (44%), and hip (40%) [26]. Women of older age and those who had previous recurring injuries prior to pregnancy were at increased risk for experiencing pain while running during pregnancy [26]. Additionally, multiparity was associated with increased odds of running-related pain specifically at the pelvis/sacroiliac joint, hip, knee, and lower back when compared to those who were pregnant for the first time [26].

### Resistance Training

A small prospective cohort study of pregnant women who underwent supervised low-to-moderate intensity strength training found no reported musculoskeletal injuries [27]. Among 675 pregnant recreational athletes who participated in heavy resistance training (defined as weightlifting at least 80% of one-repetition maximum) in a retrospective cross-sectional study, 66% did not experience any complications during pregnancy or delivery and only a minority of participants experienced unspecified injury [28].

## Pregnancy-Related Hip and Pelvic Conditions

### Pelvis

#### Clinical Presentation

The pelvic girdle consists of three bones and three joints: the fused ilium, ischium, and pubis bones, the symphysis pubis, and the two sacroiliac joints [29]. During pregnancy, ligamentous laxity results in the loosening and expansion of these joints in preparation for childbirth, and the enlarging gravid uterus shifts the center of gravity, leading to anterior pelvic tilt and compensatory lumbar hyperlordosis [5, 9]. These changes lead to pain experienced posteriorly between the posterior iliac crest and the gluteal fold near one or both sacroiliac joints and/or anteriorly at the pubic symphysis, referred to as pregnancy-related pelvic girdle pain [5, 9, 30, 31]. Pain is intermittent in nature, and can be triggered by changing of body position or prolonged sitting and standing [9, 30]. It often radiates into the groin, perineum, anterior thigh, or posterior thigh [5, 9, 30]. Onset of pain usually starts around 18 weeks of gestation, and peaks at 24–36 weeks [5]. Symptoms can be associated with reduced hip range of motion as well as altered gait [5, 30]. While pelvic girdle pain often coexists with low back pain, it is thought to be a distinct entity [31]. Discussion of pregnancy-related low back pain is beyond the scope of the current review.

Traumatic injuries to the pelvis can occur during pregnancy [1]. Pelvic ring fractures in pregnant women can occur with high-energy accidents such as motor vehicle accidents or falls from height [1]. When traumatic pelvic fractures occur, there is high risk of injury to nearby organs, including the uterus and placenta, as well as major blood vessels [1]. Pelvic and acetabular fractures are linked with high rates of complication for the mother and the fetus: death of mother (9%), intrauterine fetal demise (35%), and fetal skull fractures (18%) if the trauma occurs in the third trimester [1, 32]. All orthopedic trauma, regardless of how minor, is associated with worse pregnancy outcomes and must be assessed and treated urgently [33].

Nontraumatic pelvic fractures may also arise during pregnancy [1]. Due to exaggerated anterior tilting of the pelvis during pregnancy, pregnant women may experience sacral stress fractures [1]. Fractures of the pubic bone and sacrum that occur during labor have also been reported. According to one study that included 77 participants, pelvic fractures may occur in nearly 40–50% of women with complicated vaginal deliveries that involve factors like high birth weight or prolonged active labor [1, 34]. Peripartum pubis symphysis diastasis, while uncommon, is characterized by the separation (> 10 mm) of the pubic symphysis joint and ligaments upon delivery [1, 35]. Patients with pubic symphysis diastasis experience postpartum pubic symphysis pain and may experience immobilization if x-ray or ultrasound diagnosis is delayed [35].

#### Incidence

The incidence of pregnancy-related pelvic girdle pain varies widely in the literature, ranging from less than 10% up to 76% based upon the definition used and study design, with larger prospective studies using objective measures for diagnosis reporting a prevalence ranging from 16 to 25% [5, 30, 31, 36]. Pubic symphysis dysfunction occurs in 1 out of 3 pregnant women [43]. It is more likely to present in later trimesters of a pregnancy and arises in the third trimester 52% of the time, although it can manifest earlier, with 12% of affected women experiencing this pain in the first trimester and 34% in the second trimester [37, 38].

#### Risk Factors

Pelvic girdle pain is most strongly associated with strenuous work, low back pain, prior pregnancy-related pelvic girdle pain, and pelvic trauma [5, 9, 30, 31]. Sacroiliac joint dysfunction is more common among pregnant women who carry a large fetus, experience intense contractions, require forceps during delivery, are multiparous, and/or have rapid or precipitous labor [39]. Family history, carrying multiple subsequent pregnancies, prior back or pelvic trauma, and a sedentary lifestyle, among other factors, can make a woman more likely to experience pubic symphysis dysfunction, a disorder characterized by pain in the pubic symphysis regions, as well as diastasis, or separation, of the pubic symphysis joint [38].

#### Diagnosis

Peripartum musculoskeletal pelvic girdle pain can be identified through clinical examination or through imaging [30]. The pubic symphysis and sacroiliac joints may be tender to direct palpation [30, 31]. Provocative tests for pubic symphysis and sacroiliac joint pain include the posterior pelvic pain provocation test, Patrick’s Faber test, active straight leg raise, long dorsal sacroiliac ligament test, Gaenslen’s test, and modified Trendelenburg’s test [5, 9, 30, 31]. During pregnancy, MRI is the preferred imaging modality, though pelvic ultrasonography has been used to evaluate and monitor pubic symphyseal pain as well [5, 9, 30, 31]. Postpartum radiographs may be considered and would include AP pelvis, pelvic inlet and outlet, and flamingo views [5, 30]. Certain pelvic fractures such as stress fractures may require advanced imaging including MRI to diagnose [1].

#### Treatment

Several options for treating pelvic girdle pain are available while awaiting delivery, though they have varied results [40]. Bed rest can be considered in addition to symptomatic management with antenatal acetaminophen, cyclobenzaprine, and opioids in severe cases [5, 9, 30, 40]. NSAIDs can be used postpartum for pain relief [30]. Pelvic or sacroiliac belts may provide extra support to pregnant women, and have been shown in multiple small randomized clinical trials to help alleviate the pain in the pubic symphysis or sacroiliac joint regions [40–43]. The evidence is limited to support use of transcutaneous electrical nerve stimulation (TENS) [30, 44]. Exercise-based physical therapy including core and pelvic stability exercises are an effective treatment option for postpartum lumbopelvic pain and may be beneficial prepartum as well [31, 45]. Evidence in support of physical therapy for pregnancy-related pelvic girdle pain is limited, and if pursued should be individualized [31, 36, 46]. Imaged-guided intraarticular injections of corticosteroids and local anesthetics may be successful at reducing pubic symphysis and sacroiliac joint pain [30, 31, 40]. Epidural steroid injections may be considered in those with lumbar nerve root compression [9]. The roles of prolotherapy, radiofrequency ablation, and surgery by way of decompression or fusion remain limited [9, 30, 31, 40].

While information regarding the safety of administering corticosteroid injections to pregnant women is limited, a recent narrative review on the role of musculoskeletal injections in pregnancy concluded that current evidence suggests there are no maternal or fetal adverse effects from the use of local, non-systemic corticosteroid injections during pregnancy [47, 48].

It is worth noting that pharmacological interventions have limitations when treating musculoskeletal conditions in pregnant women to reduce potential fetal exposure to toxins. Non-steroidal anti-inflammatory drugs should be avoided by pregnant women to limit risk of congenital anomalies, major birth defects, miscarriage, preterm birth, and damage to fetal circulation [49, 50]. Paracetamol (or acetaminophen) is considered safe for use during pregnancy [44]. Opioid use should be avoided due to potential fetal toxicity [44].

Acupuncture, manual therapies (massage, chiropractic manipulation, and osteopathic manipulative therapy), and yoga are all examples of complementary and alternative medicine (CAM) therapies that can potentially alleviate low back pain and reduce disability during pregnancy, but their effectiveness needs to be further evaluated in the setting of pelvic pain and pelvic musculoskeletal conditions [31, 44, 51–53]. Acupuncture is not known to cause significant harm to a pregnant woman or the fetus [53–58]. Of note, some have proposed the presence of so-called forbidden acupuncture points among pregnant women located in the lower abdomen and sacral areas: CV3–CV7 and BL27–34 respectively [57]. However, other controlled clinical trials or observational studies demonstrated that acupuncture at these forbidden points shows no association with miscarriage or adverse pregnancy outcomes [57]. In general, with permission from a patient’s obstetrician, acupuncture can be safely implemented among pregnant women for the treatment of pregnancy-related pelvic pain during or following pregnancy [58].

Stable pelvic fractures such as stress fractures often are treated nonoperatively including offloading and rest [59]. Traumatic, unstable pelvic fractures are typically treated surgically including external pelvic stabilization and pelvic packing techniques [59].

#### Prognosis

Sacroiliac joint pain and pubic symphysis dysfunction related to pregnancy typically resolve spontaneously in the first 3 to 6 months following delivery for the majority (> 90%) of women [5, 38, 39]. Of note, recurrence is common in subsequent pregnancies [5, 30, 36].

### Hip

Several conditions affect the hip joint during pregnancy or in the postpartum period and include transient osteoporosis of the hip (TOH) and acetabular labral tears.

### Transient Osteoporosis of the Hip

#### Clinical Presentation

TOH is an uncommon, but well-described condition that typically affects women in their third trimester of pregnancy [8]. TOH is characterized by sudden onset, severe, atraumatic onset of hip pain [60, 61]. Symptoms are usually unilateral, although bilateral hip involvement has also been reported [5]. Additionally, patients may present with groin or referred anterior thigh pain, limited hip range of motion, and difficulty with weight bearing [5, 60, 62].

#### Incidence

TOH is a rare condition [61, 63]. In one study, it accounted for 2.5% of patients presenting with hip pain and is more common in middle aged males than in pregnant females [60, 64].

#### Risk Factors

Pregnancy is the primary risk factor for TOH [60, 62]. The exact cause remains unknown, but a possible explanation includes maternal skeletal resorption that occurs in the third trimester of pregnancy, as calcium is transferred away from the mother to facilitate growth of the fetus’ bones [51, 65]. This leads to a decrease in bone mineral density that can persist during breastfeeding as well [60, 62]. Some women may have a history of low bone mineral density prior to pregnancy that predisposes their risk [62]. Other proposed causes include a weight gain during pregnancy that compresses the left common iliac vein, resulting in venous hypertension, increased intraosseous pressure of the hip, and microfractures preferentially affecting the left hip [62].

One retrospective single site study found associations between TOH and older maternal age, smoking, low body mass index (BMI), family history of osteoporosis, and use of in vitro fertilization for conception [62]. Studies disagree on the effect of BMI on development of TOH, with some studies associating TOH with low BMI, and others showing an increased mean weight of 2 kg in females with TOH compared to those without [66]. Other proposed risk factors for TOH are immobility during pregnancy, dental issues during childhood, and sedentary lifestyle during childhood [61].

#### Diagnosis

Magnetic resonance imaging (MRI) is regarded as the gold standard to diagnose TOH given its sensitivity to detect changes as early as 48 h after symptom onset [5, 60, 62, 63]. MRI can show ill-defined bone marrow edema in the femoral head and neck regions, as well as in the acetabulum (hyperintense T2 weighted and hypointense T1 weighted) [5, 8, 60, 63]. A small joint effusion may also be seen [5]. Radiographs are not recommended during pregnancy and can demonstrate osteopenia of the hip 1–2 months after symptom onset [5, 60, 67]. When complicated by late-stage avascular necrosis or subchondral femoral head fracture, deformity and flattening of the femoral head can be seen on radiographs [5, 67].

#### Treatment

Pain can be treated with analgesics and hot packs [60, 62]. Off-loading the affected hip with the use of crutches, walker, and/or wheelchair is recommended for treatment to reduce pain and to protect the vulnerable joint [60, 62, 68]. Calcium supplementation can be considered [68]. Bisphosphonates are generally not recommended in pregnant women due to risks to the fetal skeletal development, however, calcitonin may reduce symptom duration and does not appear to cross the placenta [5, 8, 68–70]. Teriparatide has also been proposed as a pharmacological treatment option [60]. While TOH typically self-resolves postpartum without complication, in rare instances of disease progression where femoral head collapse or fracture occurs, surgical treatment with total hip arthroplasty or internal fixation, respectively, are options [71, 72].

#### Prognosis

Prognosis of TOH is generally good as the condition typically self-resolves 6–12 months following delivery and cessation of breastfeeding [5, 60]. Complications of TOH include fracture and avascular necrosis, especially in cases that remain undiagnosed or are diagnosed late [5, 8, 65].

### Acetabular Labral Tears

#### Clinical Presentation

Pregnancy-related acetabular labral tears present similarly to labral tears in non-pregnant patients and are characterized by groin pain, limited hip range of motion, and pain that is worse with sitting and activities [73, 74].

#### Incidence

Incidence is difficult to quantify as acetabular labral tears are common in asymptomatic patients of childbearing age [75, 76]. Nonetheless, acetabular labral tears are a known cause of postpartum hip pain [77].

#### Risk Factors

Labral tears of the hip can occur when an individual has an anatomical predisposition such as femoroacetabular impingement syndrome (FAIS) or hip dysplasia [73, 77].

#### Diagnosis

Physical exam findings for labral tears include a limp, pain with log roll of the hip, pain with flexion-adduction and internal rotation (FADIR), and pain with axial loading of the hip [73, 74]. Patrick flexion-abduction and external rotation (FABER) test can elicit groin pain in those with labral tears, and Trendelenburg sign may be positive [74, 77]. Radiographs of the pelvis and hip can show bony morphology associated with labral tears including femoroacetabular impingement lesions and hip/acetabular dysplasia [74, 77]. MR arthrogram delineates the labral tear and can indicate if there are any associated chondral injuries [74].

#### Treatment

Treatment can be non-operative or operative. Non-operative options include pain medications, activity modification, physical therapy, and intra-articular hip joint injections while operative treatment involves hip arthroscopy, labral repair or debridement, and osteochondroplasty to reshape and address any bony deformity [73, 77]. In arthritic cases, total hip arthroplasty can be considered [73].

#### Prognosis

Literature is limited on the prognosis of acetabular labral tears specifically in the pregnant or postpartum patient. There are no established guidelines regarding when to undergo elective surgery for hip pain in the context of pregnancy, and little data exists on how previous hip arthroscopy may affect pregnancy outcomes. One single-surgeon cross sectional study evaluated the perceptions of reproductive-age female patients undergoing hip arthroscopy for FAIS and found that about half of the patients were concerned that their hip pain could worsen during future pregnancy [78]. However, only a minority (15%) cited hip pain as a factor in their decision to delay or avoid pregnancy, and in general, patients considered hip arthroscopy to be safe with regard to future pregnancy outcomes [78]. This study suggested that early surgical treatment of FAIS may improve hip range of motion and may allow for easier position for laboring patients and additionally may reduce pain during sexual intercourse caused by hip positioning in flexion [78].

### Coccyx

#### Clinical Presentation

Coccydynia is a condition that affects the coccyx, the most inferior bone of the spine consisting of 3 to 5 segments, and can cause pain in pregnant and postpartum women, ranging from mild to severe enough to interfere with daily life [79–81]. Pain occurs at the tip of the coccyx and adjacent soft tissues with sitting, particularly on flat or firm surfaces, and may be exacerbated by defecation and intercourse [79–81]. Physical examination demonstrates tenderness to palpation over the coccyx [81]. Pain can have a significant impact on quality of life, and may be associated with urogynecological, rectal and sexual dysfunction [79, 80].

#### Incidence

Incidence is variable, ranging from 4 to 15% of women in the postpartum period [79].

#### Risk Factors

Trauma, or a fall directly onto the coccyx, is the most common etiology of coccydynia in all patients [80]. Postpartum women are susceptible to the development of coccydynia due to the mechanical stress to the sacrococcygeal joint during labor [79–81]. Risk factors include female sex, age of 30–50 years, hypermobility of the coccyx, anatomic morphology of the coccyx, short perineum, complicated vaginal delivery, and obesity [79–81]. One study found that postpartum coccydynia was more commonly associated with difficult and/or instrumented deliveries such as with forceps or vacuum [80, 82].

#### Diagnosis

In the majority of patients, coccydynia is primarily a clinical diagnosis. Imaging with radiographs (including AP and lateral views) is an important initial diagnostic step to evaluate for fracture, dislocation, or predisposing morphology of the coccyx, including retroverted coccyx, a bony spicule, or coccygeal subluxation, and to rule out rare etiologies such as infection or tumor [80]. In addition, dynamic imaging may be used to evaluate for coccygeal hypermobility or hypomobility, including seated and standing radiographs [80]. Computed tomography (CT) or MRI may also be helpful in the evaluation of a patient with coccydynia but have found to be inconclusive in a majority of patients [80]. In the postpartum patient, it is important to evaluate for other etiologies of pain in this region, including pelvic floor dysfunction.

#### Treatment

Treatment is centered on pain management with oral analgesics, heat and/or cryotherapy, and ergonomic modifications, including avoidance of sitting positions that exacerbate pain and offloading the coccyx with a donut or ring-shaped cushion [79, 83]. Other interventions, such as steroid injections or prolotherapy, nerve blocks (ganglion impar block), manual therapy (massage and/or manipulation), physical therapy, or transcutaneous electrical nerve stimulation can be sought out if conservative management strategies are unsuccessful [79–81, 83]. Image-guided corticosteroid injections, typically in the sacrococcygeal joint or around the sacrococcygeal ligaments can alleviate the symptoms of refractory cases of coccydynia, and can also be useful diagnostic tool to identify patients who may benefit from coccygectomy [80, 81, 83]. Finally, surgical coccygectomy may be considered in patients refractory to all other conservative treatment options [80, 81].

#### Prognosis

The literature is sparse with regard to prognosis of coccydynia in pregnant and postpartum patients. In the general population, coccydynia often resolves spontaneously, and conservative management has been reported to be successful in up to 90% of patients [80].

### Nerve Conditions

Nerve conditions, such as meralgia paresthetica and sciatica, are other musculoskeletal conditions associated with pregnancy. The majority of studies in pregnant women group the discussion of sciatica with low back pain, making it challenging to discuss them separately.

### Meralgia Paresthetica

#### Clinical Presentation

Meralgia paresthetica (MP) is a peripheral sensory neuropathy that occurs in the distribution of the lateral femoral cutaneous nerve (LFCN). The LFCN is a purely sensory nerve that arises from the lumbar nerve roots, most commonly L2 and L3, and traverses under the inguinal ligament, medial to the anterior superior iliac spine, to supply sensory innervation to the anterolateral proximal thigh [84–86]. Patients present with numbness, paresthesia, and/or pain over the anterolateral, proximal thigh. Physical examination is notable for a localized area of pain and/or altered sensation in the distribution of the LFCN. As this is a purely sensory nerve, motor function, reflexes, and muscle bulk are preserved [85–87].

#### Incidence

The incidence of MP in the general population is reported to range from approximately 3 to 4 per 10,000 person-years, with twelve times higher odds in pregnancy [85, 88]. The specific incidence in pregnancy has not been reported.

#### Risk Factors

This condition typically arises from mechanical compression under the inguinal ligament or stretching of the LFCN during pregnancy and labor, with risk factors including gestational diabetes, high weight gain of the mother, excessive growth of the fetus, and prolonged second stage of labor [84].

#### Diagnosis

MP can be clinically diagnosed through physical exam, including a pelvic compression test performed [85, 86]. The condition can be confirmed through a nerve conduction study if necessary, and is safe to perform during pregnancy [84]. A diagnostic injection of local anesthetic around the LFCN can also help confirm the diagnosis of MP [86].

#### Treatment

During pregnancy, treatment focuses on symptom management [85–87, 89]. Treatment options for MP include avoiding compression of the nerve by offloading the front of the hip and thigh in the inguinal region with avoidance of tight-fitting clothing and aggravating positions that may compress the nerve [84, 85, 87]. In addition, ultrasound-guided nerve blocks, transcutaneous electrical nerve stimulation, lidocaine patches (US Food and Drug Administration, category B), kinesiology taping, and active release techniques can be considered [84, 85, 87]. Surgical treatment with neurolysis and/or neurectomy is reserved for refractory cases [85, 89].

#### Prognosis

The majority of cases either resolve spontaneously within a few months after delivery, and/or improve with non-operative modalities [84–87].

### Sciatica

#### Clinical Presentation

Sciatica is a neuropathy involving the sciatic nerve or nerve roots (L4-S3) and presents with radicular pain and/or paresthesia in the sciatic nerve distribution, classically originating in the lumbar spine or buttocks and radiating down the posterior thigh to the lateral calf and plantar foot [90]. On physical examination, patients may have a positive straight leg raise test or Lasègue’s sign, as well as a positive crossed straight leg raise test [90, 91].

#### Incidence

Reports of low back pain during pregnancy vary widely in the literature, ranging from 25 to 90% of women, with most studies estimating that around half of pregnant women will experience low back pain during pregnancy [92–95]. This pain should be differentiated from true sciatica, which arises when a herniated disc compresses the lumbar nerve roots or with compression of the sciatic nerve around the pelvis, which is only reported in 1% of pregnant women [93].

#### Risk Factors

Pregnancy itself, due to enlarging uterus size, shifting center of gravity, and hormonal influences resulting in ligamentous laxity, may be risk factors for development of sciatica [94, 96]. Use of forceps during delivery, prolonged labor, and prolonged stretching or compression of the sciatic nerve while in the lithotomy position may contribute to the development of sciatica [94]. Sciatica may also be associated with use of epidural anesthesia during delivery [87, 94].

#### Diagnosis

Sciatica can be diagnosed clinically with a thorough history and physical examination, as described above [90, 91]. Advanced imaging with MRI is indicated in the presence of true neurologic deficit, “red flag” symptoms suggestive of infection or malignancy, or in patients with severe or progressive symptoms not responsive to conservative management for 6–8 weeks [90–92]. MRI may detect a herniated intervertebral disc and nerve root compression, and can help rule out more serious etiologies of sciatica such as infection or tumor [90, 91, 94].

#### Treatment

Conservative treatment should start with pain management with analgesics, heat and cryotherapy, postural modifications and avoidance of positions that exacerbate pain, gentle stretching, light physical activities, and physical therapy [87, 90, 91, 95]. Existing evidence supports implementation of eight-to 12-week exercise programs to treat low-back and pelvic pain in pregnancy, as well as osteomanipulative therapy for low-back pain and acupuncture for pelvic pain [36]. Epidural steroid injections can be considered, as these are thought to be likely safe in pregnancy, although long-term safety data are lacking in the literature [87, 92]. Surgery, including discectomy and/or foraminotomy, is typically avoided in pregnancy unless there are significant progressive neurologic deficits, severe intractable radicular pain refractory to conservative treatment, or cauda equina syndrome [87, 92]. Delivery of the fetus, when appropriate, may also result in resolution of sciatica, and the optimal mode of delivery in women with lumbar disc herniation is debated in the field, with some authors advocating for elective cesarian Sects. [87, 92, 94].

#### Prognosis

The prognosis in pregnant patients appears to be similar to the general population where sciatica often resolves with non-surgical interventions and typically improves following delivery [91, 95].

## Prevention of Pregnancy-Related Hip and Pelvic Musculoskeletal Conditions

Physical activity and exercise are important means by which women can reduce their risk of pregnancy-related hip and pelvic musculoskeletal conditions. The American College of Obstetricians and Gynecologists (ACOG) recommends that active women continue their pre-pregnancy level of non-contact physical activity [97]. Because a moderately active lifestyle does not place women at higher risk of musculoskeletal injury, it is advised that women perform light to moderate intensity activities to preserve musculoskeletal system health [25, 97]. The U.S. Department of Health and Human Services recommends that women achieve at least 150 min of moderate intensity aerobic activity per week during pregnancy and the postpartum period [98]. Women who previously participated in vigorous intensity aerobic activity prior to pregnancy can continue those activities during pregnancy [98]. A large body of literature indicates that the following types of exercise are both safe and beneficial for women during pregnancy: walking, stationary cycling, aerobic exercise, dancing, resistance training, stretching, and water aerobics [97]. In addition, light to moderate weight training has not been associated with adverse maternal or fetal effects during pregnancy, but data on strenuous strength training during pregnancy is sparse [98, 99].

A two-armed, multicenter randomized control trial of 855 pregnant women studied the effects from the combination of low-impact moderate intensity aerobic exercise, body weight resistance exercises, and balance exercises [100]. This 12-week exercise program was implemented from 20 to 36 weeks of gestation. The authors found no difference between intervention or control groups regarding incidence of lumbopelvic pain at 36 weeks; however, the intervention group had a significantly lower proportion of participants who required sick leave due to lumbopelvic pain [100]. Similarly, a smaller randomized control trial of 257 pregnant women found no difference in the incidence of low back pain or pelvic girdle pain between the control and intervention groups [101]. The intervention in this study included supervised group exercises consisting of light aerobics, body weight resistance exercises, and stretching [34].

A randomized control trial of 516 pregnant women found that those who underwent unsupervised water exercises twice weekly starting from approximately 20 weeks of gestation experienced significantly lower intensity of low back pain compared to the control group at follow up [102]. However, the authors questioned the clinical significance of their results as there were no differences between groups for the number of sick days, disability from low back pain, or self-perceptions of general health [102]. Another smaller randomized control trial of 54 pregnant women revealed significantly lower levels of musculoskeletal pain in the water exercise group compared to the control group [103].

Evidence is limited regarding the use of physical activity and exercise specifically to reduce the risk of hip and pelvic musculoskeletal problems. A Cochrane systematic review from 2015 concluded there is low- to moderate-quality evidence supporting that exercise during pregnancy may reduce the number of women who report low back and pelvic pain, as well as pain-related functional disability and sick leave [55]. A more recent systemic review and meta-analysis from 2018 of 32 randomized controlled trials found “very low” to “moderate” quality evidence that exercise during pregnancy did not reduce the odds of low back, pelvic girdle and lumbopelvic pain during pregnancy or in the postpartum period, but did reduce the severity of these conditions during pregnancy and postpartum [104]. Other more recent systematic reviews on this topic have similarly concluded there is no clear evidence for the protective effects of prenatal exercise on pelvic girdle pain or lumbopelvic pain. There is inconsistent evidence on the effects of regular physical activity before pregnancy and the risk of developing low back pain and/or pelvic girdle pain during pregnancy [99, 105, 106]. Owe and colleagues found that women who exercised regularly and engage in high-impact physical activities prior to pregnancy had a lower risk of developing pelvic girdle pain in pregnancy compared to non-exercisers [107, 108]. The inconsistent findings and low quality of evidence in these studies highlights the need for additional high quality randomized controlled trials to evaluate the types of physical activity and exercise interventions that best prevent the development of hip and pelvic musculoskeletal conditions in pregnant women.

Other areas of primary prevention may include bracing or orthotics, weight control, and sleeping position. A Cochrane review found low quality evidence that wearing a rigid pelvic support belt during exercise did not enhance the pain-relieving effects of exercise on pelvic pain, and found that a non-rigid lumbopelvic belt reduced pelvic pain and functional disability more than exercise, for up to 6 weeks after treatment [53]. More recent studies have called into question the effectiveness of maternity support belts. Bey et al. found only very small changes in postural stability noted in wearers [109]. A systematic review on this topic found insufficient evidence supporting the use of maternity support belts to reduce pregnancy-related low back pain and/or pelvic girdle pain, with potential adverse effects including skin irritation, pain, and potential fetal heart rate changes [110]. Finally, while there is ample literature linking maternal obesity and excessive weight gain in pregnancy to adverse maternal and infant outcomes, the literature does not directly correlate weight control with prevention of musculoskeletal pain [44].

## Limitations

Our review highlights that existing studies that examine musculoskeletal conditions among pregnant women are limited by biases that accompany self-reported data, small sample sizes, and observational study designs. These limitations in the quality of existing data call for higher quality randomized control trials, especially those focused on pre-habilitation programs, physical activity guidelines, and guidance regarding the safety of medications and alternative treatment modalities.

## Conclusion

It is our recommendation that obstetricians, orthopaedic surgeons, and sports medicine physicians come to consensus regarding the diagnostic criteria for each type of disorder, as many disorders in the regions encompassing the pelvis and hip share similar symptoms. Distinguishing between the similar symptoms of these disorders could yield increased clarity regarding effective prevention and treatments for pregnant patients. Further research on pre-habilitation programs, physical activity guidelines, and guidance regarding the safety of medicines and alternative treatment modalities would be helpful for pregnant patients.

As we continue to learn about the etiology of musculoskeletal conditions during pregnancy, we can shift our focus to providing evidence-based recommendations and guidance to providers and pregnant patients. When women are provided specific physical activity recommendations in preparation for pregnancy, they may feel empowered and better prepared to face musculoskeletal issues that may arise both during and following pregnancy.

## Data Availability

No datasets were generated or analysed during the current study.

## Key References

- M. Thabah and V. Ravindran, “Musculoskeletal problems in pregnancy,” *Rheumatol. Int.*, vol. 35, no. 4, pp. 581–587, Apr. 2015, doi: 10.1007/s00296-014-3135-7.

This reference is a comprehensive review that details the physiological and musculoskeletal changes that accompany pregnancy. It provides a clear outline of these musculoskeletal changes and distinguishes between true hip pain and hip pain that originates from the back or sacroiliac regions.

- N. K. Kanakaris, C. S. Roberts, and P. V. Giannoudis, “Pregnancy-related pelvic girdle pain: an update,” *BMC Med.*, vol. 9, no. 1, p. 15, Dec. 2011, doi: 10.1186/1741-7015-9-15.

This reference focuses on pelvic girdle pain and attempts to resolve lack of consensus regarding the prognosis of pregnancy-related pelvic girdle pain by examining previous literature, risk factors, and diagnosis, among other factors.

- F. Fiat et al., “The Main Changes in Pregnancy—Therapeutic Approach to Musculoskeletal Pain,” *Medicina (Mex.)*, vol. 58, no. 8, p. 1115, Aug. 2022, doi: 10.3390/medicina58081115.

This reference promotes use of less invasive alternative methods in place of pharmacological treatments for musculoskeletal pain in pregnancy, which supports advocacy for pre-habilitation exercises and programs.

### Conflict of interest

Stephanie E. Wong is the editor-in-chief of Current Reviews in Musculoskeletal Medicine and has no financial disclosures. All other authors have no financial or non-financial interests related to this work.
